# Determinants and inequality of recurrent aphthous stomatitis in an Indonesian population: a cross sectional study

**DOI:** 10.1186/s12903-023-03683-8

**Published:** 2023-12-19

**Authors:** Herry Novrinda, Catherine Salsabila Azhara, Anton Rahardjo, Atik Ramadhani, Han Dong-Hun

**Affiliations:** 1 Department of Dental Public Health and Preventive Dentistry, Universitas Indonesia, Jakarta, Indonesia; 2 Department of Preventive and Social Dentistry, Seoul National University School of Dentistry, Seoul, Korea; 3 Dental Research Institute, Seoul National University, Seoul, Korea

**Keywords:** Aphthous stomatitis, Ulcer, Socioeconomic disparities in health, Health surveys, Indonesia

## Abstract

**Introduction:**

Recurrent Aphthous Stomatitis (RAS) is the most common type of ulceration in the oral cavity which can occur due to several factors.

**Aims:**

To determine the factors related to the incidence of RAS and examine the social class inequality in RAS among the Indonesian population using data from the fifth wave of the Indonesian Family Life Survey (IFLS5) in 2014.

**Methods:**

This study is a descriptive study with a cross-sectional design using secondary data from IFLS5 data in 2014 (n = 28,410). Socio-economic position (SEP) was calculated by Adult Equivalent Scale and categorized into 4 classes. Outcome is RAS. Mediating factors were psychosocial (stress), eating behavioral (food consumption), and systemic diseases. Descriptive analysis, chi-square and a series of logistic regressions were performed to analyze the data. Odds ratio (OR) and 95% confidence interval (CI) were used to report the results.

**Results:**

Only 18.39% of IFLS5 respondents experienced the incidence of recurrent aphthous stomatitis in 2014. The bivariate (chi-square) results showed that there was a relationship between the incidence of RAS and the related independent variables. Logistic regression showed the highest possibility of RAS in respondents aged 18–34 years, female, unmarried, high school education level, living in the city, having frequent stress levels, having food habits that frequently drinking soda, sweet foods, chili sauce, fried food and has systemic diseases such as, asthma, cancer, rheumatism, and digestion. The lowest SEP group had the highest probability of occurrence of RAS over the other groups among the different models.

**Conclusions:**

There are several factors that determine the incidence of RAS. There was a monotonic gradient of inequality in RAS according to SEP group. This study might be useful to provide information regarding the relationship of determinants factors with the incidence of RAS to prevent it and promote oral health in the future.

## Introduction

Thrush or recurrent aphthous stomatitis (RAS) is the most common type of ulceration in the oral cavity [1]. It is often referred to as *canker sores* by patients and healthcare providers [2]. RAS is reported to affect up to 25% of the population which characterized by painful, round, or oval shaped ulcers with an inflammatory halo [3]. RAS is one of the most common oral disease in Indonesia, with a prevalence of 12% [4].

Several predisposing, local, immune, microbial, and other factors have been reported as triggers for RAS [5], but the exact cause of this disease remains unclear. A previous study showed that RAS could occur due to several factors [1] with a higher predilection for females (51.6%) than males. The incidence of aphthous ulcers may be associated with history of systemic diseases as finding in Iranian population [6]. Additionally, RAS is reported to be associated with the eating habits (consumption of certain foods or drinks) and stress levels of the individual [7]. A positive family history and gastrointestinal problem are also reported to have a relationship with RAS [8]. A previous study evaluated the association between RAS and depression in the Indonesian population using data from the Indonesia Family Life Survey (IFLS-4) [4].

However, to the best of our knowledge, there is no study on RAS and its determinant factors in relation to social class inequality in the Indonesian population. Therefore, the aims of this study were to (1) determine the factors related to the incidence of RAS and (2) examine the social class inequality in RAS among the Indonesian population using data from the IFLS-5 in 2014.

## Materials and methods

### Study design and sample

This cross-sectional study comprised data from the fifth IFLS conducted by RAND Labor and Population in 2014 and 2015 (https://www.rand.org/well-being/social-and-behavioral-policy/data/FLS/IFLS/ifls5.html). The IFLS is a repeated cross-sectional survey regarding socioeconomic and health, in which individual household members are interviewed at different times; the survey has been conducted five times (1993, 1997, 2000, 2007, and 2014) in Indonesia. The information was collected at the individual, household, and community levels, and sampling was performed using the simple random sampling method. The IFLS comprised both nominal and ordinal data. The original sample framework for the current study was based on households from 13 of Indonesia’s 33 provinces containing 83% of the population: four provinces on Sumatra (North Sumatra, West Sumatra, South Sumatra, and Lampung), five of the Javanese provinces (DKI Jakarta, West Java, Central Java, DI Yogyakarta, and East Java), and four provinces covering the remaining major island groups (Bali, West Nusa Tenggara, South Kalimantan, and South Sulawesi). The data obtained in IFLS5 is only in the form of questionnaires and interviews (subjective) without intraoral examination. The aspects of measured oral health were toothache and RAS [9].

### Variables

RAS is a subjective condition; therefore, it was measured using a Yes/No question based on symptoms that occurred over the past 4 weeks. The participants were categorized based on their age (in years) as follows: 18–34, 35–54, 55–64, and > 65. The gender options were Male or Female, whereas the educational status comprised the following categories: none/ no-school; elementary school; junior high school/secondary; senior high school/tertiary; and college/university. Information about marital status (unmarried; married; divorced), area of residence (urban or rural), and smoking status were obtained. Additionally, information about the presence of psychological problems, the level of stress, systemic disease, and dietary habits were collected from the survey. The socioeconomic status (SES) was measured using the Adult Equivalent Scales (AES), and the participants were divided into four groups (SES 1 [lowest AES] to SES 4 [highest AES]). Banks et al. defined AES as the proportional increase in income per adult required to maintain a certain standard of living due to various changes in demographic circumstances [10]. The AES can be used to compare the welfare of households with different conditions or arrangements. The measurement of living standards can be used to divide groups based on the SES. The following formula can be used to calculate the number of Adult Equivalents (AE) in a household:

$$AE = {(A + aK)^\theta },$$

Where A represents the number of adults in the household, K represents the number of children, and a represents the cost of children aged 0–14. According to previous study, “a” is worth 0.5 in Indonesia because it is classified as a developing country, and θ is the measurement scale used; the economic scale of a family was 0.75 for families with more than one member [10–12].

### Statistical analysis

Descriptive statistics, the Chi-square test, and logistic regression analyses were conducted using STATA 17 (*StataCorp LLC, USA*) to analyze the IFLS-5 data. The characteristics of respondents were presented by the RAS with frequency distributions for the categorical variables. The Chi-square test for categorical variables was used to assess the relationships between RAS and other variables. The models were serially adjusted for age (continuous), gender, stress, eating habits, and systemic disease using logistic regression analyses. SES 4 was assigned as the reference group, and the baseline model (model 1) was adjusted for age and gender.

## Results

The total number of respondents in this study was 28,410, and 5,244 (18.39%) experienced RAS. Most of the IFLS-5 respondents (45.03%) were in category of aged 18–34 group with range 18–101 year old and *X* ± SD (38.85 ± 14.14), married (76.76%), female (53.76%), high school (31.48%) and lived in urban areas (59.17%). The distribution of respondents based on the history of systemic diseases was as follows: hypertension (12.50%), diabetes (2.35%), tuberculosis (0.95%), asthma (2.82%), heart disease (1.61%), stroke (0.63%), liver (1.03%), cancer (0.66%), rheumatism (5.12%), kidney disease (1.41%), and digestion (13.06%) with the incidence of RAS. Generally, the most common systemic diseases experienced by the participants were related to the digestive system (13.06%). Fried foods, chili sauce, sweet foods, and soda drinks were frequently consumed by 21.57%, 45.07%, 15.98%, and 1.41% of the participants, respectively.

As seen in Tables 1 and 2, several variables, such as age, marital status, gender, educational attainment, area of residence, SES, smoking habit, stress level, and the consumption of fried/ oily snacks, chili (spicy) sauce, sweet foods, and soda, were significantly associated with RAS. Several systemic diseases/conditions, such as hypertension, tuberculosis, asthma, cancer, rheumatism, and digestive diseases, were also significantly associated with RAS.

**Table 1:** Association between demographic, smoking, psychosocial variables, and RAS among Indonesian population in IFLS-5

Variables		Having RAS		Free of RAS	*P*-value*
	*n*	%	*n*	%	
**Age (years old)**					
18–34	2483	20.5%	9658	79.5%	**< 0,001**
35–44	1258	18.6%	5506	81.4%	
45–54	695	15.5%	3798	84.5%	
55–64	417	15.1%	2336	84.9%	
> 64	214	13.3%	1393	86.7%	
**Marital Status**					
Married	4010	18.1%	18,087	81.9%	**< 0,001**
Ever Married / Separated	149	19.1%	633	80.9%	
Not Married Yet	828	20.8%	3151	79.2%	
**Gender**					
Female	2924	19.1%	12,348	80.9%	**< 0,001**
Male	2300	17.5%	10,838	82.5%	
**Educational Attainment**					
None / Not in School	216	14.5%	1278	85.5%	**< 0,001**
Elementary School	1529	17.4%	7245	82.6%	
Middle School	1011	19.2%	4261	80.8%	
High School	1753	19.6%	7191	80.4%	
Collage / University	715	18.2%	3211	81.8%	
**Area of Residence**					
Rural	1950	16.8%	9650	83.2%	**< 0,001**
Urban	3274	19.5%	13,536	80.5%	
**SES Groups**					
SES 1 (Lowest)	885	16.0%	4632	84.0%	**< 0.001**
SES 2	1071	17.6%	5014	82.4%	
SES 3	1097	18.9%	4701	81.1%	
SES 4 (Highest)	2171	19.7%	8839	80.3%	
**Smoking**					
Not Smoking	3492	19.6%	14,365	80.4%	**< 0,001**
Smoking	1732	16.4%	8821	83.6%	
**Depression Experience**					
No	5216	18.4%	23,152	81.6%	0.912
Yes	8	19.0%	34	81.0%	
**Stress**					
Rarely/Never	3470	16.6%	17,425	83.4%	**< 0.001**
Few (1–2 days)	839	22.8%	2834	77.2%	
Sometimes (3–4 days)	638	23.0%	2134	77.0%	
Often (5–7 days)	277	25.9%	793	74.1%	

*Obtained from Chi-Square test

**Table 2:** Association between behavioral, systemic diseases, and RAS among Indonesian population in IFLS-5

Variables		Having RAS		Free of RAS	*P*-value*
	*n*	%	*n*	%	
**Fried / oily Snack**					
Never or Rarely	2341	16.8%	11,580	83.2%	**< 0.001**
Sometimes	1627	19.5%	6734	80.5%	
Often	1256	20.5%	4872	79.5%	
**Chili (Spicy) Sauce**					
Never or Rarely	1368	16.6%	6877	83.4%	**< 0.001**
Sometimes	1388	18.9%	5973	81.1%	
Often	2468	19.3%	10,336	80.7%	
**Consumption of sweet food**					
Never or Rarely	2884	16.9%	14,192	83.1%	**< 0.001**
Sometimes	1375	20.2%	5420	79.8%	
Often	965	21.3%	3574	78.7%	
**Drinking Soda**					
Never or Rarely	4639	17.9%	21,263	82.1%	**< 0.001**
Sometimes	490	23.3%	1617	76.7%	
Often	95	23.7%	306	76.3%	
**Systemic Disease**					
**Hypertension**					
No	4499	18.1%	20,360	81.9%	**0.001**
Yes	725	20.4%	2826	79.6%	
**Diabetes**					
No	5104	18.4%	22,639	81.6%	0.789
Yes	120	18.0%	547	82.0%	
**Tuberculosis**					
No	5159	18.3%	22,982	81.7%	**< 0.014**
Yes	65	24.2%	204	75.8%	
**Asthma**					
No	5037	18.2%	22,571	81.8%	**< 0.001**
Yes	187	23.3%	615	76.7%	
**Heart / Cardiac**					
No	5124	18.3%	22,811	81.7%	0.131
Yes	100	21.1%	375	78.9%	
**Stroke**					
No	5190	18.4%	23,041	81.6%	0.834
Yes	34	19.0%	145	81.0%	
**Liver / Hepar**					
No	5173	18.4%	22,945	81.6%	0.683
Yes	51	17.5%	241	82.5%	
**Cancer**					
No	5175	18.3%	23,047	81.7%	**0.006**
Yes	49	26.1%	139	73.9%	**< 0.000**
**Rheumatism**					
No	4877	18.1%	22,078	81.9%	**< 0.001**
Yes	347	23.8%	1108	76.2%	
**Digestive**					
No	4247	17.2%	20,392	82.8%	**< 0.001**
Yes	977	25.9%	2794	74.1%	
**Renal /Kidney**					
No	5142	18.4%	22,868	81.6%	0.272
Yes	82	20.5%	318	79.5%	

*obtained from Chi-Square test

Table 3 shows that the OR of RAS was negatively correlated with age. Female sex, lower SES, higher stress, frequent consumption of spicy/chili sauce, fried/oily, sweet food, and soda, and several systemic diseases presented with high ORs for RAS.

**Table 3:** Logistic Regression of Selected Determinants with RAS among Indonesian Population in IFLS-5

Variables	OR (95%CI)**
**Age**	
18–34	1
35–44	0.87 (0.81–0.94)
45–54	0.70 (0.64–0.77)
55–64	0.68 (0.61–0.76)
> 64	0.59 (0.50–0.68)
**Gender**	
Male	1
Female	1.11 (1.05–1.18)
**Economic Status**	
SES 1 (Lowest)	1.17 (1.06–1.28)
SES 2	1.10 (1.01–1.20)
SES 3	1.04 (0.96–1.13)
SES 4 (Highest)	1
**Stress**	
Rarely/Never (< 1 day)	1
Few (1–2 days)	1.48 (1.36–1.61)
Sometimes (3–4 days)	1.50 (1.36–1.65)
Often (5–7 days)	1.75 (1.52–2.02)
**Smoking Status**	
No Smoking	1
Smoking	0.80 (0.75–0.86)
**Chili/spicy Food**	
Never or Rarely	1
Sometimes	1.14 (1.03–1.25)
Often	1.15 (1.07–1.22)
**Fried/Oily Food**	
Never or Rarely	1
Sometimes	1.17 (1.07–1.27)
Often	1.22 (1.14–1.32)
**Sweet Food**	
Never or Rarely	1
Sometimes	1.23 (1.12–1.35)
Often	1.28 (1.18–1.39)
**Drinking Soda**	
Never or Rarely	1
Sometimes	1.28 (1.08–1.51)
Often	1.39 (1.10–1.76)
**Asthma**	
No	1
Yes	1.36 (1.15–1.60)
**Cancer**	
No	1
Yes	1.56 (1.13–2.17)
**Rheumatism**	
No	1
Yes	1.41 (1.25–1.60)
**Digestion**	
No	1
Yes	1.67 (1.54–1.81)

**P value < 0.0001 for all variables

The ORs of each SES and the explanatory powers of the mediating factors that explain the association between SES and the occurrence of RAS are shown in Table 4. The lowest SES group had the highest probability of developing RAS, followed by the low-middle group. The explanatory power for psychosocial factors (stress) was the highest in all four SES groups. The change in OR due to psychosocial factors was 6.65%, 1.17%, and 8.19%, respectively, in Model 2. In Model 4, systemic diseases presented with the second highest explanatory power with a 20.29%, 23.21%, and 33.24% change in OR in the lowest, mid-low, and middle groups, respectively.

**Table 4:** Explanatory power of potential mediating factors in the association between SES and Occurrence of RAS among Indonesian population

SES	Model 1	Model 2	*Δ*OR	Model 3	*Δ*OR	Model 4	*Δ*OR	Model 5	*Δ*OR
SES 1 (Lowest)	**1.23 (1.12–1.34)**	**1.24 (1.14–1.35)**	6.65	**1.15 (1.05–1.25)**	34.57	**1.18 (1.08–1.29)**	20,29	**1.13 (1.03–1.23)**	43,92
SES 2	**1.13 (1.05–1.23)**	**1.14 (1.05–1.23)**	1.17	**1.09 (1.00-1.18)**	35.31	**1.10 (1.02–1.20)**	23.21	1.07 (0.98–1.16)	51.49
SES 3	1.048 (0.967–1.137)	1.052 (0.970–1.141)	8.19	1.02 (0.94–1.11)	51.78	1.03 (0.95–1.12)	33.24	1.01 (0.93–1.10)	70.17
SES 4 (Highest)	1.00 (reference)	1.00 (reference)		1.00 (reference)		1.00 (reference)		1.00 (reference)	

Model 1 was adjusted for age and gender.

Model 2 was adjusted for age, gender (model 1) plus psychosocial factors (Stress).

Model 3 was adjusted for age, gender (model 1) plus Food Consumption Behaviors (Drinking soda, Chili sauce, Fried/oily, Sweet-food).

Model 4 was adjusted for age, gender (model 1) plus Systemic Diseases (Asthma, Cancer, Rhematic, Digestive)

Model 5 was adjusted for age, gender (model 1) plus all factors

*Δ*OR = (OR_model *x*_*-* OR_model 1_/ OR_model 1_*–* 1)*100%

**Bold denotes statistical significance at*p*** **< 0.05**

## Discussion

Nearly one-fifth (18.39%) of the Indonesian population in this study experienced RAS. There is an increasing of proportion of the population with RAS in Indonesia from IFLS-4 (11.95%) [4]. This should be of concern to all relevant parties in Indonesia because increasing an oral disease prevalence has a multiplier effect [13]. As reported previously, females and those belonging to the younger age group appeared more prone to RAS [14]; this might be related to the onset of the menstrual cycle, pregnancy, and dysmenorrhea [15].

Stress is hypothesized to raise salivary cortisol and enhance immunoregulatory action by increasing the amount of leukocytes at the site of inflammation [16]. Stress was acknowledged as a direct contributor of RAS even among dental students [17]. Another previous study reported a significant association between stress during the COVID-19 pandemic and the occurrence of RAS in Indonesian college students [18]. Aphthous stomatitis was also proposed as one of potential oral disease related to climate anxiety and stress [19]. Surprisingly, the depression was not significantly associated with RAS in the current study; this is not in accordance with the findings of a previous study related to RAS in Indonesia, [4] which could be attributed to differences in the type of data; the previous study used the IFLS-4 (2007) data, whereas the IFLS-5 (2014) data were used in this study.

Another interesting finding was that smokers had a lower probability of getting SAR than non-smokers. This finding was in line with the findings in several previous studies regarding smoking status on the incidence of SAR [3, 20–22]. Alcohol use and smoking might be protective factors against SAR as revealed in a study among Turkish adults [3]. There is a negative relationship between smoking and the incidence of SAR among Polish population. SAR recurrence occurred more frequently in the non-smoker group [21]. The mechanism by which smokers are more resistant to SAR has been attempted to explain, including that this occurs due to protective hyperkeratinization of the oral mucosa. RAS is only found on nonkeratinized oral mucosa and not on keratinized oral mucosa. This could be due to the considerable difference in microbiota composition between keratinized and nonkeratinized mucosa [23]. In addition, smoking is closely related to changes in the oral microbiota, which may contribute an opposite effect to the pathogenesis of RAS [22]. Salivary epidermal growth factor (EGF) has been shown to be beneficial in the treatment and prevention of recurring aphthous ulcers. Smokers’ increased salivary epidermal growth factor would be an intermediary circlet for smoking’s effect on the formation of RAS [20]. These findings are not intended to justify smoking habits, but rather to encourage scholars to further investigate the relationship between smoking and the incidence of RAS in order to reach more accurate conclusions.

Consumption of spicy food, fried/oily snacks, sweet foods, and soda drinks was significantly associated with the prevalence of RAS in the Indonesian population. Consistent with the findings of a previous study, [24]. RAS was closely linked to several systemic diseases, including digestive disorders. This information should be considered when implementing a common risk factor approach (CRFA) [25] in relation to RAS. The consumption of spicy and oily foods and soda is known a risk factor for gastritis [26]. Therefore, Efforts to promote health and prevent RAS can be coordinated with those to prevent other diseases, such as gastritis.

The lower SES groups had higher ORs than those in the highest SES group. The status of those in the SES 1 and SES 2 groups remained even after adjusting for the various variables. The socioeconomic condition, which was reflected by the AES, appeared to be predominantly correlated with RAS in this study, indicating that income and/or expenditure play an important contributory role in inequality in oral health [27]. The SES affects health; [28] as the finding in a previous study using IFLS-5 regarding significant association between health condition (wasted) and SES [29]. A recent systematic review and meta-analysis reported that individuals of low SES had poorer OHRQoL, regardless of the economic classification of the country [30]. A previous study among Indonesian migrant workers in South Korea also highlighted the impact of SES in oral health [31]. According to the IFLS-5 data, the oral health of the Indonesian population is closely related to the SES. Thus, oral health efforts should also be closely related to efforts to improve people’s welfare both from the government and from the people themselves. Furthermore, health promotion, prevention, and treatment efforts should consider the SES of the individual and the community.

A limitation of this study is that conclusions about the causality of RAS cannot be made owing to the cross-sectional nature of the study. The use of secondary data allows for the processing of data that is already available. Although oral health subjective condition (RAS) is deemed reliable [32], subjectivity in the responses of participants is likely given that the variables were only collected via interview. Nonetheless, this study comprised data representative of the national health data; hence, it can be used as a reference for national health policies. The identification of the determinants of RAS will aid in focusing on the promotion and intervention efforts in the right direction. This is the first study to examine the role of RAS and its determinants in relation to the SES in the Indonesian population.

## Conclusions

Several determinants, such as SES, eating habits, and systemic diseases, were correlated with the occurrence of RAS in the Indonesian population. A monotonous gradient of inequality in RAS according to the SES group. A lower SES may increase the probability of RAS in the Indonesian population even after adjusting for various determinants. The findings of this study will contribute to our knowledge about the relationships between SES and the various determinants of RAS and aid in preventing this disease in the future.

## Data Availability

This cross-sectional study comprised data from the fifth IFLS conducted by RAND Labor and Population in 2014 and 2015. Availability online at (https://www.rand.org/well-being/social-and-behavioral policy/data/FLS/IFLS/ifls5.html).

## List of Abbreviations

AE: Adult Equivalents
AES: Adult Equivalent Scales
IFLS: Indonesia Family Life Survey
RAS: Recurrent aphthous stomatitis
SES: Socio-economic status
SEP: Socio-economic position
UGM: University of Gadjah Mada
